# Supplementary material for: Contextualizing Parental/Familial Influence on Physical Activity in Adolescents before and during COVID-19 Pandemic: A Prospective Analysis
Source: Children (Basel). 2020 Sep 3;7(9):125. doi: 10.3390/children7090125 (PMC7552694; doi:10.3390/children7090125)
Supplement: Supplementary file 1 [file children-07-00125-s001.pdf]

Supplementary table S1

Frequencies (F) and percentages (%) in studied sociodemographic-, parental- and familial factors with differences between boys and girls (MW – Mann Whitney test)

	Boys		Girls		MW	
	F	%	F	%	MW	p
**Socioeconomic status of the family**					0.39	0.69
Below average	12	3.3	2	0.6		
Average	328	89.6	308	95.7		
Above average	26	7.1	12	3.7		
**Paternal education**					3.37	0.001
Elementary school	16	4.4	36	11.2		
High school	257	70.2	226	70.2		
College	47	12.8	36	11.2		
University level	46	12.6	24	7.5		
**Maternal education**					3.33	0.01
Elementary school	84	23.0	120	37.3		
High school	238	65.0	160	49.7		
College	24	6.6	20	6.2		
University level	20	5.5	22	6.8		
**Parental conflict**					5.45	0.001
Never	174	47.5	92	28.6		
Rarely	134	36.6	142	44.1		
From time to time	54	14.8	72	22.4		
Often/regularly	4	1.1	16	5.0		
**Parental absence from home**					0.83	0.40
Never	55	15.0	80	24.8		
Rarely	107	29.2	70	21.7		
From time to time	138	37.7	106	32.9		
Often/regularly	64	17.5	66	20.5		
**Parental questioning**					2.37	0.02
Never	12	3.3	8	2.5		
Rarely	54	14.8	32	9.9		
From time to time	160	43.7	96	29.8		
Often/regularly	140	38.3	184	57.1		
**Parental care**					4.54	0.001
Very poor	6	1.6	0	0.0		
Low	10	2.7	10	3.1		

Parents care about me	141	38.5	100	31.1
Parents care about me a lot	209	57.1	212	65.8
